# A virtual simulation-based training program on birthing positions: a randomized controlled trial

**DOI:** 10.1186/s12912-023-01491-7

**Published:** 2023-09-15

**Authors:** Huimin Lin, Guihua Liu, Xiaoyan Wang, Qin Xu, Shengbin Guo, Rongfang Hu

**Affiliations:** 1 The School of Nursing, Fujian Medical University, Fuzhou City, China; 2 Fujian Maternity and Child Health Hospital, College of Clinical Medicine for Obstetrics & Gynecology and Pediatrics, Fuzhou City, China

**Keywords:** Virtual simulation, Midwife, Position, Kirkpatrick, In-service training

## Abstract

**Background:**

Restricting parturient women in healthcare facilities from choosing positions that provide the greatest comfort and benefit during labor is a global barrier. Several complex factors, including caregiver preference and medical intervention, shape the limitation. Therefore, a practical need exists to train midwives on the knowledge and skills to change this condition.

**Methods:**

The study used a parallel, single-blind, randomized controlled trial at a provincial maternity and child health hospital in Fujian, China, from June 1 to December 31, 2019. The midwives in a birth suite were selected and randomly enrolled in a one-month simulation-based hybrid training or face-to-face teaching in September 2019. The four-level Kirkpatrick’s model, including *reaction, learning, behavior, and results*, was used to evaluate training effects before and after the program. Data were analyzed with SPSS 25.0 using Student’s *t*-test, Spearman’s correlation test, Mann–Whitney U test, Wilcoxon signed-rank test, and chi-square test analysis of variance. The significance level was set at *p* < 0.05.

**Results:**

Forty-two midwives were initially randomized to either the virtual simulation group or the face-to-face group. One midwife was excluded from the analysis due to intervention discontinuation, resulting in a final analysis of 41 midwives (n1 = 21, n2 = 20). Post-intervention, the virtual simulation group exhibited higher satisfaction and learning effects compared to the face-to-face group, while the rate of perineal incision in primiparas was lower (*p*<0.05). No significant changes or differences were observed in self-rated behavior between the two groups (*p*>0.05). The virtual simulation group demonstrated an increase in non-supine birth rate (*p* = 0.030) and a decrease in perineal incision rate among primiparas compared to pre-intervention (*p* = 0.035). Moreover, knowledge performance was associated with the duration of virtual simulation (*r* = 0.664, *p* = 0.001).

**Conclusions:**

Virtual simulation is a fascinating innovation that enables midwives to develop birthing positions without practicing on real pregnant women and is one solution to achieve work competency within a shortened training period.

## Introduction

Birthing positions affect the biomechanics and physiologic adaptions to labor. The World Health Organization [1] provided *recommendations on intrapartum care for a positive childbirth experience*, which suggests the possibility of choosing any position, except the supine position, for the woman to be most comfortable throughout labor and birth. It is widely recognized that the supine position is associated with adverse maternal outcomes, including a higher incidence of cesarean section, instrumented vaginal births, perineal trauma, episiotomies, and postpartum hemorrhage [2, 3]. By contrast, upright or lateral positions could benefit women by relaxing ligaments, taking weight off the sacrum, and allowing flexibility of the pubic symphysis. Uterine contractions can be enhanced and gain efficiency in dilating the cervix, resulting in a shorter duration of pushing time [4]. Expansion of the pelvic outlet can reduce biomechanical complications, such as shoulder dystocia, in newborns [5, 6]. Furthermore, adopting maternal upright positions, such as kneeling, standing, sitting, and squatting, can provide comfort to most women, while also reducing the risk of compressing the mother’s aorta, which is the primary source of oxygen and nutrition for the baby [4]. These findings were supported by a systematic review [7].

However, several studies indicated that the medicalization of childbirth was accompanied by birth attendants who disregarded women’s preferences and chose positions that they considered convenient for medical intervention and monitoring [8]. The evidence points to a harsh reality in healthcare facilities where women are commonly restricted to supine positions. As such, it is crucial to incorporate the provision of information and benefits related to birthing positions into respectful maternity care, particularly for low-risk women, and to encourage them to adopt their preferred positions.

As the primary caregiver of perinatal maternal and infant health, midwives often exert significant influence over women’s decision-making regarding birthing positions [8]. However, research has demonstrated that midwives may not be sufficiently flexible or willing to implement alternative positions, even though these were preferred by women [9, 10]. For changes to occur from the caregivers’ perspective and within the healthcare system, in-service training should be offered to midwives to acquire skills and confidence.

The utilization of interactive technology in virtual simulation enables the recreation of reality in computer-generated environment, providing trainees with the opportunity to make decisions within a low-risk setting [11]. This innovative pedagogical approach serves as a complementary strategy to learning in practical settings. Foronda C et al. [12] discovered that this training method can be applied for low-morbidity and high-risk diseases, enabling interns to practice events they may not encounter in clinical practice. Additionally, Virtual simulation can be integrated into a comprehensive curriculum either face-to-face or online [13]. A nursing program that utilizes this hybrid training has identified its positive aspects, such as promoting flexible learning and enhancing student confidence and engagement [14]. Virtual simulation interventions have been shown to improve learners’ learning outcomes (skills-based, cognitive, and affective), compared to the face-to-face approach [15]. The use of virtual simulation technology is valuable in the safe and effective development of health professionals’ abilities. In this regard, we developed a 3-Dimensional (3-D) virtual medical app, named the “Birthing Positions Training System,“ which offers various observation modes, such as cross-section and 360-degree rotation, to support technology-enabled learning and online assessment methods, thereby overcoming the limitations of time and space.

The Kirkpatrick Model, developed by Donald Kirkpatrick in the 1950s, comprises four sequential levels: *reaction, learning, behavior and results* [15]. This model is renowned for its comprehensive evaluation approach, which has been extensively employed for nearly six decades to assess the effectiveness of training programs [16]. Furthermore, it is utilized to establish and evaluate short-term and long-term outcomes [16]. Therefore, the Kirkpatrick Model would be a wise choice for the hybrid training evaluation programs.

The aim of this study was to assess the effectiveness of virtual simulation-based training in comparison to traditional face-to-face training for midwives, utilizing the Kirkpatrick model. Notably, there is a dearth of literature on the application of virtual simulation technology in the training of birthing positions. Therefore, this study sought to address this research gap and enhance the knowledge and skills of midwives.

## Methods

### Study design

This study adopted a parallel, single-blind, randomized controlled trial with a pre- and post-test design. A repeated-measures design was employed to evaluate the effects of the training program using Kirkpatrick’s model.

### Participants and setting

This study was conducted at Fujian Maternity and Child Health Hospital in Fujian, China, which boasts an annual birth rate exceeding 20,000, during the period spanning from June 1 to December 31, 2019. The study employed the following inclusion criteria: (1) employment in the birth suite; (2) possession of a nurse license and a certificate of competency in maternal and child health care; (3) provision of informed consent for voluntary participation in the study. Conversely, the exclusion criteria were as follows: (1) nursing students; (2) trainee midwives; (3) receipt of birthing positions training within the past year.

The sample size was determined using a one-tailed, independent *t*-test, a power of 0.80 with an alpha of 0.05, and an effect size of d = 0.80. Using G*Power 3.1.9.4 [17], the required sample size was 42, with 21 in each group.

A computer-generated randomization method randomly assigned subjects to the experimental or control group. The randomization allocation sequence was concealed from the researchers in sequentially numbered opaque envelopes that were sealed and stapled. Envelopes were opened by a naive third party, a Research Assistantship (RA), unaware of the participant’s condition. The RA did not participate in the training.

**Fig. 1:**
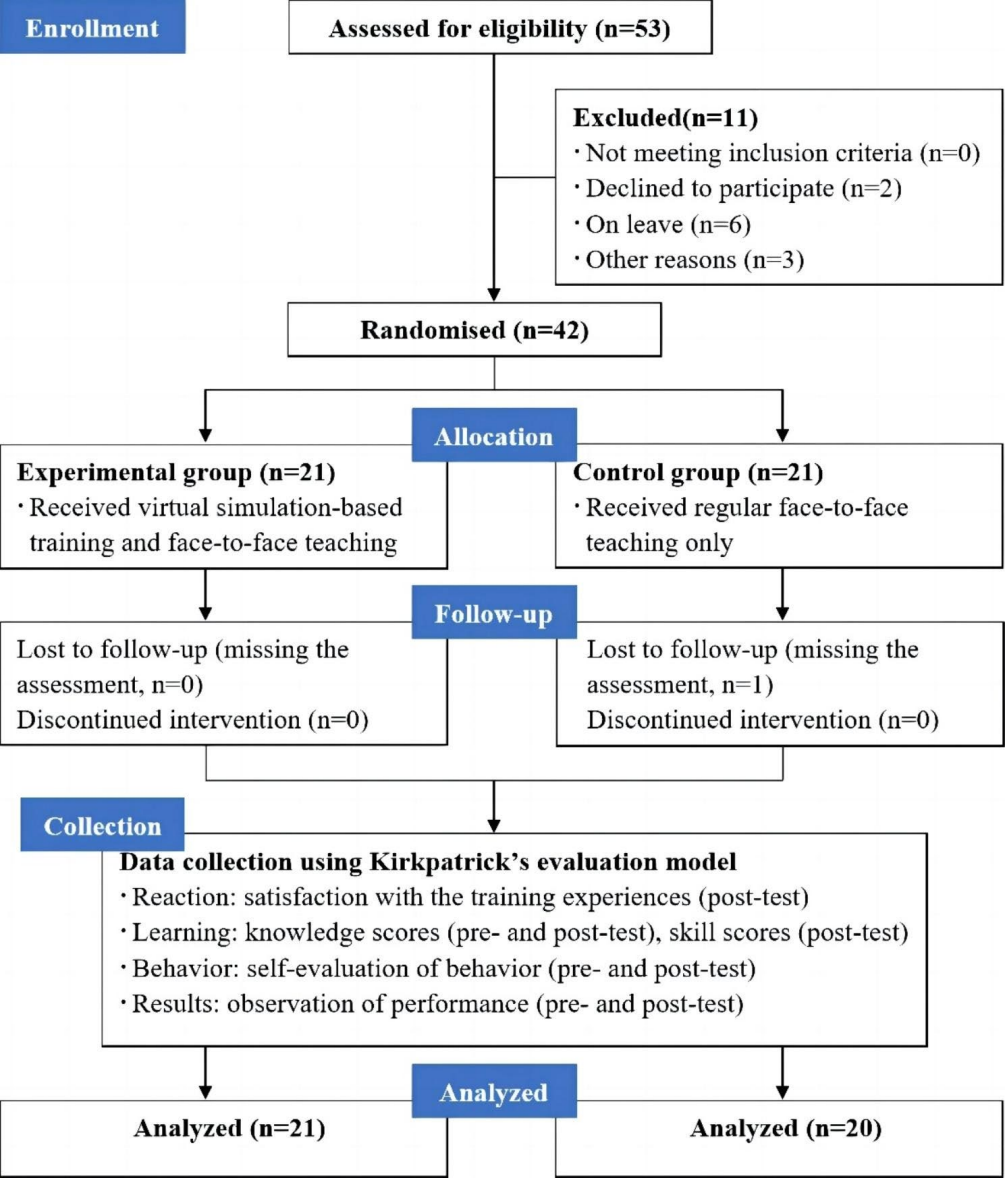
Flow diagram of study design

Invitations, either written or oral, were extended to 53 midwives who fulfilled the established criteria. Six midwives were unable to attend due to leave, while three were unable to attend for personal reasons. Additionally, two midwives declined the invitation. One participant in the face-to-face group dropped out midway due to personal leave. Ultimately, 41 participants provided informed consent and successfully completed the training program. Figure 1 depicts the flow diagram of the participants.

### Ethics

The study was conducted according to the Declaration of Helsinki and approved by the ethics committee of a university (no.:2017-48; date:2017/10/24); Institutional approval was obtained from the hospital management. A research team member gave all participants an oral and written explanation of the study’s objectives and procedure. The Birthing Positions Training System did not collect individual identities at the control terminal. Voluntary participation and data confidentiality were also ensured. Those who participated in the training program received a ¥100 gift after completing the final evaluation to acknowledge their contributions.

### Interventions and procedures

#### Materials

The study protocol was formulated by expert group meetings and was strictly followed during the research. The training was based on the Positions and Movements section of the evidence-based textbook entitled The Labor Progress Handbook [18]. The research team members developed, modified, and pre-tested the pre- and post-questionnaires. Cronbach’s alpha measured the internal consistency of the questionnaires for reaction (0.962) and behavior (0.933). Factor analysis showed that structural validity indices were all showed greater than 0.5.

**Fig. 2:**
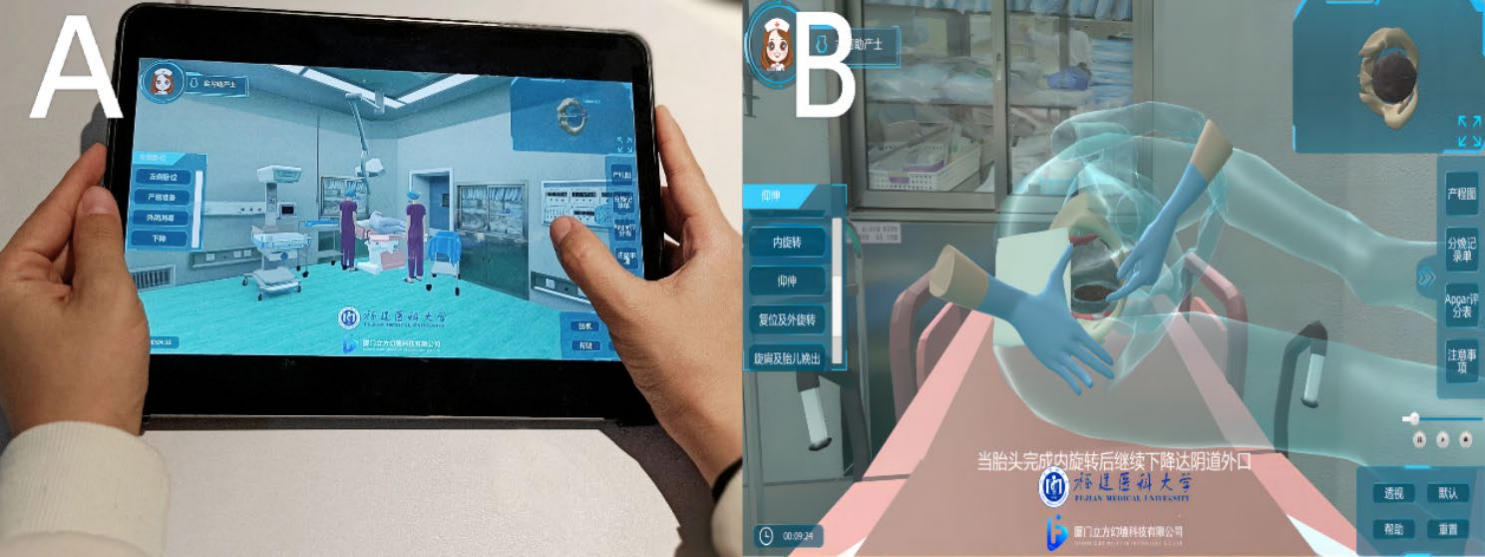
App screenshot of the Birthing Positions Training System. It presents the first person’s viewpoint of a midwife who controls the midwife avatar in a 3D birth suite virtual environment. The midwife can perform a health assessment and positions guide on the patient’s avatar by clicking on the body parts or control panel

Meanwhile, we developed the Maternal Position Training System and obtained the Chinese software copyright (copyright number: 2019SR0729250). Based on clinical cases of normal birth, the application (*app*) created a “real-life” labor ward setting as a prototype with characters based on midwives and mothers. In addition, the user terminal could display the changes in the maternal spine, pelvic diameter, uterus, and fetus under different birthing positions, such as standing, sitting, lying, kneeling, and squatting. Figure 2 shows a screenshot of the app.

#### Instructors

The in-person training was all conducted by a midwife who possessed more than 25 years of position experience. The virtual simulation group also received technical assistance from two professors on app using issues.

#### Interventions

The virtual simulation group executed a hybrid training program spanning one month, wherein a textbook, training schedule, app installation package, and app tutorial were disseminated. The midwives were instructed to utilize the app and complete weekly homework assignments within it. Three-hour in-person training sessions were conducted by an instructor every second week, in conjunction with the computer version of the Birthing Positions Training System. The Birthing Positions Training System was utilized to demonstrate abstract content, including the principal mechanism of positions. Furthermore, a nearly one-hour in-person debriefing was arranged in the last week of training for midwives to share their clinical position practice experience.

In contrast to the virtual simulation group, the face-to-face group underwent a conventional one-month face-to-face training regimen. The midwives were furnished with textbooks and training schedules. In addition, they were required to partake in self-directed learning and submit written assignments on a weekly basis. The in-person training was also conducted every second week and lasted for three hours each, incorporated live lectures, group practice, and on-site instruction. Physical teaching aids, such as birthing balls and birthing mats, were employed to demonstrate the skills of birthing positions in real-time, while textbooks and pictures were utilized to elucidate the position mechanisms. Likewise, the face-to-face group conducted a field experience sharing in the last week of training.

### Outcome measures and instruments

The training impact was assessed using Kirkpatrick’s model of four evaluation levels. The instrument used to assess each level is discussed below.

The first evaluation level (*Reaction*) considered midwife participation and the degree of their reactions to the training activity organization and content. In this study, a self-administered questionnaire, Satisfaction Assessment Questionnaire, was conducted online to evaluate their satisfaction level and perceived assessment of the training benefits at its end. The questionnaire includes eight items, including textbook, training schedule, instructional apparatus, instructor, training method, learning atmosphere, personal initiative, and training effect.

The second evaluation level (*Learning*) assessed the training’s effect. The pre- and post-test mainly assessed the midwives’ degree of knowledge about birthing positions. An online examination consisting of 20 multiple-choice questions (4 points per item) and 10 true or false questions (2 points per item) was administered. Following the training, we analyzed the proficiency of the midwives’ skills by Objective Structured Clinical Examination (OSCE). Two third-party personnel used a series of pre-designed test stations simulating clinical scenarios to comprehensively evaluate the midwives’ clinical skills and humanistic care in applying the positions. The test content collects and evaluates six stations: *medical history, lying, sitting, standing, kneeling*, and *squatting*. We used the official hospital rating scale to evaluate the skills of both groups, with a total score of 100.

The third evaluation level (*Behavior*) assessed whether the participants’ learning was transformed into practice using the Behavior Self-Evaluation Questionnaire three months before and after the intervention. The questionnaire consisted of 12 items rated on a 5-point Likert scale. The questionnaire comprises eight items, encompassing topics such as first health-assessment, dynamic assessment, women need, family participation, normative behavior, risk identification, risk response, communication, initiative, respect, and patience.

The last evaluation level (*Results*) measured the changes the training has brought to a given organization. A checklist was administered to compare the impacts of both training programs on childbirth outcomes in mothers and newborns three months pre- and post-training. The data was extracted from the electronic health record system, pertaining to the non-supine birth rate, episiotomy rate, degree of perineal lacerations, incidence of bleeding greater than 400 mL at 2 h postpartum, and low Apgar score rate within 1 min after birth.

### Data collection

Two naive third parties, who have over 10 years’ professional experience in birthing positions, used five tools to collect data pertinent to the study: the Satisfaction Assessment Questionnaire, Position Knowledge Test, OSCE of Position, Behavior Self-Evaluation Questionnaire, and Checklist of Results. Online questionnaires were delivered to the participants three times: pre-training (T0), post-training immediately (T1), and three months post-training (T2). First, the Position Knowledge Test, Behavior Self-Evaluation Questionnaire, and Checklist of Results were conducted at T0 to compare the baseline. Next, the Satisfaction Assessment Questionnaire, Position Knowledge Test, and OSCE of Position were used at T1. Finally, the Behavior Self-Evaluation Questionnaire and Checklist of Results were administered at T2. Except for the OSCE, which lasted two days, the rest of the data collection was done within a day.

### Data analysis

All statistical analyses of the collected data were conducted using IBM SPSS Statistics for Windows, Version 25.0 (Armonk, NY: IBM Corp). We calculated descriptive statistics (median, quartile, means, standard deviations, frequency, and frequency ratio) to describe the response distributions for the four levels. We used a Student’s *t*-test to assess the knowledge and manipulative skill performance between or within both groups and a Spearman’s correlation test to assess the relationship between the length of online learning and performance. We also applied a Mann–Whitney U test and Wilcoxon signed-rank test to compare item scores and a chi-square test to juxtapose birthing positions. Statistical significance was set at *p* < 0.05.

## Results

### Participant characteristics

Forty-one midwives ultimately participated in this study (n1 = 21 in the virtual simulation group and n2 = 20 in the face-to-face group). All the midwives were female. Their ages ranged from 25 to 45 years. Table 1 presents the baseline characteristics, which were equivalent between the two groups.

**Table 1:** Participants’ baseline characteristics (N = 41)

Characteristics	Virtual simulation (n = 21)	Face-to-face (n = 20)	Test statistics	*p value*
Age (years)			-1.218^a^	0.223
≤ 29	12(57.14)	9(45.00)		
30 ~ 34	7(33.33)	5(25.00)		
35 ~ 39	2(9.52)	5(25.00)		
≥ 40	0(0.00)	1(5.00)		
Marital Status			0.042^b^	0.871
Married	8(38.10)	7(35.00)		
Unmarried	13(61.90)	13(65.00)		
Education Level			0.061^b^	0.901
Junior college	8(38.10)	8(40.00)		
Undergraduate	13(61.90)	12(60.00)		
Length of Midwifery Work (years)			-0.71^a^	0.943
≤ 5	11(52.38)	10(50.00)		
6~9	6(28.57)	5(25.00)		
≥ 10	4(19.05)	5(25.00)		
Professional Title			-0.085^a^	0.932
Nurse	0(0.00)	1(5.00)		
Nurse Practitioner	19(90.48)	16(80.00)		
Nurse in Charge	2(9.52)	3(15.00)		
Health Professional Level			-0.922^a^	0.356
N1	3(14.29)	6(30.00)		
N2	7(33.33)	6(30.00)		
N3	0(0.00)	1(5.00)		
N4	7(33.33)	6(30.00)		
N5	3(14.29)	1(5.00)		

### Level 1: ***reaction***

The training satisfaction survey showed marked variation in the total score between participants in the virtual simulation group and face-to-face group (35.86 ± 4.63 vs.32.75 ± 5.05, *p* = 0.047).

### Level 2: ***learning***

As presented in Table 2, the learning results of both groups improved significantly (61.33 ± 12.98 to 75.14 ± 7.99, *p*<0.001; 56.60 ± 11.24 to 67.90 ± 7.82, *p*<0.001) from the pre-test scores. In addition, compared to the control, the experimental group had higher post-test scores in knowledge and manipulative skills after controlling for the pre-test scores (75.14 ± 7.99 vs. 67.90 ± 7.82, *p* = 0.006; 91.83 ± 3.60 vs. 88.68 ± 3.29, *p* = 0.006). The Pearson correlation results demonstrated the knowledge performance in the virtual simulation group was associated with the duration of online learning (mean duration of online time: 1.48 h; *r* = 0.664, *p* = 0.001), and no association between skill performance and online duration was found (*r* = 0.238, *p* = 0.300).

**Table 2:** Comparison of knowledge and skill scores

Groups	T0 (Mean ± SD)	T2 (Mean ± SD)	*t*	*p* value
Knowledge scores				
Virtual simulation	61.33 ± 12.982	75.14 ± 7.989	4.648^b^	0.000
Face-to-face	56.60 ± 11.244	67.90 ± 7.820	4.718^b^	0.000
	*t* = 1.219^a^, *p* = 0.230	*t* = 2.932^a^, *p* = 0.006		
Skill scores				
Virtual simulation	-	91.83 ± 3.600	-	-
Face-to-face	-	88.68 ± 3.290	-	-
		*t* = 2.928^a^, *p* = 0.006		

### Level 3: ***behavior***

The pre-test self-assessment scores of behaviors did not indicate differences between the two groups (*p* = 0.196). However, according to pre- and post-test result comparisons using a Wilcoxon signed-rank test, the virtual simulation group scores improved for defining maternal postural requirements and guiding companions to cooperate with the mother (*p* = 0.018, *p* = 0.031), whereas the face-to-face group had no significant change across all items. In addition, a Mann–Whitney U test revealed that the scores concerning the two abovementioned items were higher than those for the face-to-face group (*p* = 0.009, *p* = 0.028).

### Level 4: ***results***

According to Pearson’s chi-square test, the non-supine birth rate of the virtual simulation group increased (*p* = 0.030) in T2, while that of the face-to-face group did not change (*p* = 0.246). No difference was found between the pre- and post-test results of the two groups in the non-supine birth rate (*p*>0.05). Table 3 presents the positions development of the two groups. The rate of perineal incision of primiparas decreased in the experimental group after the training (*p* = 0.035) and was lower than that of the control group (*p* = 0.040). The face-to-face group did not exhibit a wide disparity between the pre- and post-test (*p*>0.05), and no significant difference was found between the two groups before the training (*p* = 0.079). There was no variation in the degree of perineal lacerations, the incidence of bleeding over 400 mL in the 2 h postpartum, and the rate of low Apgar scores within 1 min after birth in the primipara and multipara of the two groups at the pre- and post-test (*p*>0.05).

**Table 3:** Comparison of childbirth positions frequency

Groups	Position types	T0 Frequency (%)	T2 Frequency (%)	${\varvec{\chi }}^{2}$	*p value*
Virtual simulation	Supine position	109(8.1)	112(6.1)	4.691	0.030
	Non-supine position	1231(91.8)	1710(93.9)		
Face-to-face	Supine position	104(7.2)	106(6.1)	1.345	0.246
	Non-supine position	1347(92.8)	1620(93.9)		
		${\chi }^{2}$=0.924, *p*=0.336	${\chi }^{2}$=0.000, *p*=0.994		

## Discussion

The virtual simulation training program quickly brought benefits to the organization, accelerating the positive change in participants’ training reaction, knowledge, skills, behavior towards birthing position.

The satisfaction of midwives in the intervention group was generally higher than that of the control group. This finding was consistent with the research of Sivarajah RT et.al [19], which found that traditional teaching methods can no longer meet the needs of learners. Unlike the traditional “spoon-fed” training model, virtual simulation fully mobilizes the learners’ initiative [20]. The virtual simulation provided the operators with visual, audio, and tactile stimulation not supplied in the traditional passive learning. The literature, including our study, indicates that virtual simulation technology is more engaging and enjoyable, and can even enhance team dynamics [21]. Learners are more actively engaged and immersed in the simulation environment, which no doubt that can lead to better training outcomes. The positive response to this approach underscores the importance of incorporating enjoyable elements into training programs to optimize learner engagement.

The acquisition of knowledge and clinical skills is a vital component of medical training. Training effectiveness, according to some experts, is closely connected to instructional strategies [22]. In our study, the utilization of solely face-to-face presentation methodology does make a difference in the learning outcomes, however, the interactive influence of virtual simulation is more pronounced. Furthermore, the length of time spent engaged in virtual learning was found to be positively correlated with the acquisition of knowledge. The transformation of abstract and intricate concepts into a dynamic and visually stimulating 3D format through virtual simulation may facilitate comprehension and memory [23]. There are also no spatial or temporal restrictions using the mobile terminals, so learners can learn autonomously and repeatedly until they are satisfied. Those technical strengths contributed to virtual simulation gaining popularity [24]. As such, virtual simulation can be a valuable supplement to the regular teaching modalities.

Training outcomes have been overwhelmingly focused on knowledge gains, with less attention paid to learner behavior [25]. It’s necessary to extend the assessment of training effects to include behavioral outcomes and organizational benefits. Due to the lagged effect of the transfer of knowledge and skills on behavior [26], we evaluated behavioral changes and analyzed organizational performance improvement in different training programs three months after the training.

About half of the training effects were due to changes in the participants after they returned to work, according to Kirkpatrick [27]. In terms of behavioral performance, the midwives in the virtual simulation group exhibited a modest improvement in post-training scores compared to their pre-training scores. However, this change was not deemed significant. This finding is consistent with previous reports indicating that only a small amount of what learned in training is applied to the clinical practice in healthcare settings [28]. The transfer of knowledge, skills, and attitudes acquired during training to the workplace is a multifaceted process that involves not only the training program itself, but also the work environment, organizational support, and professional development opportunities [29]. On the other hand, intentions to change behavior were measured using self-reported measures; however, self-reported intent does not always translate into actual behavior change.

With regard to the result level, the hybrid approach appears to be a more effective means of advancing the position of the birth suite compared to conventional in-person training. Midwives use a greater variety of positions in the virtual simulation intervention context, where changes in knowledge may lead to changes in clinical practice in the long run. Furthermore, imaging evidence has verified that lateral decubitus, hands-and-knees, and sitting positions increase the pelvis diameter, reduce the episiotomy rate, and facilitate natural childbirth [30, 31]. This evidence may account for the reduced incidence of perineal incisions observed in the virtual group during this investigation. These results imply that the implementation of virtual simulation-based instruction has the potential to enhance the performance of both departments and hospitals, and generate favorable outcomes at the organizational level.

## Limitations

Several measures were adopted to reduce the likelihood of contact during the study to avoid information exchange between the two groups of midwives during training, such as sharing the research content and arrangements with the midwives before the study commenced. Participants were asked not to share the online training materials between the two groups. This strategy ensured that all training materials would be shared for the control group after the three-month follow up and that the two groups of midwives worked separately, with the consent of the department. However, this single-center study was still susceptible to confounding factors and had a small sample size, which may have affected the evaluation results of the training. Therefore, a multi-center study with a large intervention dose, long-term follow-up, and process evaluation should be conducted in the future. Furthermore, qualitative research methods can further explore a deeper understanding this interventional method.

## Conclusions

In this study, we used Kirkpatrick’s model as a training evaluation tool to comprehensively evaluate the effectiveness of the virtual simulation-based training program. Our findings indicate that the training program was a successful approach in enhancing learner response, training satisfaction, and learning outcomes. Nevertheless, the translation of training into individual behavior change is hindered by the complexity of behavior transformation or the discrepancy between self-reported intent and actual behavior change. As well, the “transfer problem” poses a challenge to organizational performance. Although the increase in non-supine positions rate and lower episiotomy rate were observed, their direct attribution to the training program remains unclear. Nonetheless, The potential of virtual simulation technology in training is undeniable and warrants further exploration.

## Data Availability

The datasets generated and/or analysed during the current study are available in the [in-service training program data] at https://gitee.com/huimin-lin/in-service-training-program-data.

## Abbreviations

3-D: 3-Dimensional
RA: Research Assistantship
App: Application
OSCE: Objective Structured Clinical Examination
